# Expression of _NES-_hTERT in Cancer Cells Delays Cell Cycle Progression and Increases Sensitivity to Genotoxic Stress

**DOI:** 10.1371/journal.pone.0010812

**Published:** 2010-05-25

**Authors:** Olga A. Kovalenko, Jessica Kaplunov, Utz Herbig, Sonia deToledo, Edouard I. Azzam, Janine H. Santos

**Affiliations:** 1 Department of Pharmacology and Physiology, New Jersey Medical School, Newark, New Jersey, United States of America; 2 Department of Pathology, New Jersey Medical School, Newark, New Jersey, United States of America; 3 Department of Microbiology and Molecular Genetics, New Jersey Medical School, Newark, New Jersey, United States of America; 4 Department of Radiology, New Jersey Medical School, Newark, New Jersey, United States of America; University of Illinois at Chicago, United States of America

## Abstract

Telomerase is a reverse transcriptase associated with cellular immortality through telomere maintenance. This enzyme is activated in 90% of human cancers, and inhibitors of telomerase are currently in clinical trials to counteract tumor growth. Many aspects of telomerase biology have been investigated for therapy, particularly inhibition of the enzyme, but little was done regarding its subcellular shuttling. We have recently shown that mutations in the nuclear export signal of hTERT, the catalytic component of telomerase, led to a mutant (_NES-_hTERT) that failed to immortalize cells despite nuclear localization and catalytic activity. Expression of _NES-_hTERT in primary fibroblast resulted in telomere-based premature senescence and mitochondrial dysfunction. Here we show that expression of _NES-_hTERT in LNCaP, SQ20B and HeLa cells rapidly and significantly decreases their proliferation rate and ability to form colonies in soft agar while not interfering with endogenous telomerase activity. The cancer cells showed increased DNA damage at telomeric and extra-telomeric sites, and became sensitive to ionizing radiation and hydrogen peroxide exposures. Our data show that expression of _NES-_hTERT efficiently counteracts cancer cell growth *in vitro* in at least two different ways, and suggest manipulation with the NES of hTERT or its subcellular shuttling as a new strategy for cancer treatment.

## Introduction

A key property of malignant tumors is their ability to proliferate indefinitely. This is mediated, in 90% of the cases, by the reactivation of telomerase, a reverse transcriptase responsible for maintaining telomeres [1], [2]. Telomerase is composed minimally of two different subunits, a catalytic core (hTERT) and an RNA component (hTR), which work in concert to replenish telomeres with each cell division. hTERT has been recently shown to acquire properties of an RNA-dependent RNA polymerase when in a complex with the RNA component of the mitochondrial endoribonuclease MRP [3]; such activity is not involved in the maintenance of telomeres. Whereas hTR is present in both somatic and germ cells constitutively, expression of hTERT is tightly regulated. Telomerase activity is high during embryogenesis and in the vast majority of tumors, but is low or non-existent in most adult somatic cells [1]. For that reason, inhibiting telomerase has become a promising strategy for cancer treatment.

Several different approaches have been developed to block the activity of telomerase holoenzyme, ranging from inhibitors of hTERT to G-quadruplex stabilizing agents to targeted degradation of the associated hTR [4]–[17]. In all cases, direct or indirect telomerase inhibition results in the inability of the cells to maintain telomeres and ultimately cells arrest growth or die. A problem of these approaches is that several weeks to months are required for the effects as they mostly rely on extensive telomere shortening [5]. Nonetheless, telomerase inhibitors are currently in clinical trials [18].

We have recently shown that a mutant hTERT defective in its nuclear export signal (_NES-_hTERT) failed to maintain telomeres and “healthy” mitochondria in both primary and SV40-transformed human fibroblasts [19]. Despite nuclear localization and catalytic activity in vitro, the mutant protein was biologically inactive in vivo leading to premature senescence with activation of the classical telomere-related DNA damage response (DDR), when expressed in primary cells. Expression of the mutant protein was also associated with mitochondrial dysfunction and DNA damage to both telomeric and extra-telomeric sites [19]. Given the rapid and dramatic effects observed, we hypothesized that ectopic expression of _NES-_hTERT may also be an effective means to counteract cancer cell growth. In the present study we expressed _NES-_hTERT in various cancer cells lines and show a rapid and efficient delay in cell cycle progression without any detectable change in the levels of endogenous telomerase enzymatic activity. Expression of the mutant protein significantly decreases the ability of the cells for anchorage-independent growth in vitro. We found that ectopic expression of _NES-_hTERT led to nuclear telomeric, extra-telomeric and mitochondrial DNA (mtDNA) damage in the cancer cells and sensitized at least one type of cancer cells to both oxidative stress and γ-radiation. Taken together, our results suggest targeting the NES of hTERT or its intracellular movement as a novel strategy to effectively counteract tumor cell growth.

## Results

### Overexpression of _NES-_hTERT in skin and prostate cancer cell lines rapidly blocks cell cycle progression

We have recently shown that ectopic expression of a mutant hTERT in which the NES has been disrupted (_NES_-hTERT) causes premature senescence in telomerase-negative human fibroblasts [19]. Primary cells expressing _NES-_hTERT stopped growing within 5-20 population doublings after introduction of the mutant protein, which was associated with classical signs of cellular senescence such as blockade in the G1 to S transition, upregulation of p21^waf1^, p16 and positivity for senescence-associated β-galactosidase (β-gal) activity [19]. Given these effects, we asked whether expression of _NES-_hTERT would also impact cell cycle progression of telomerase-positive cancer cells. To address this question, we stably expressed _NES-_hTERT in SQ20B (squamous cell carcinoma - skin cancer) and LNCaP (prostate cancer) cells and followed growth in mass cultures for several weeks; control cells were either left non-infected or infected with empty vector. No differences were observed between non-infected and empty-vector transduced cells (data not shown). Cells were selected with antibiotics for 2 weeks prior to initiation of the experiments. Viral transductions were repeated at least two independent times with each cell line showing reproducible results. All experiments presented herein rely on data obtained with cells derived from at least two independent viral infections.

**Figure 1:**
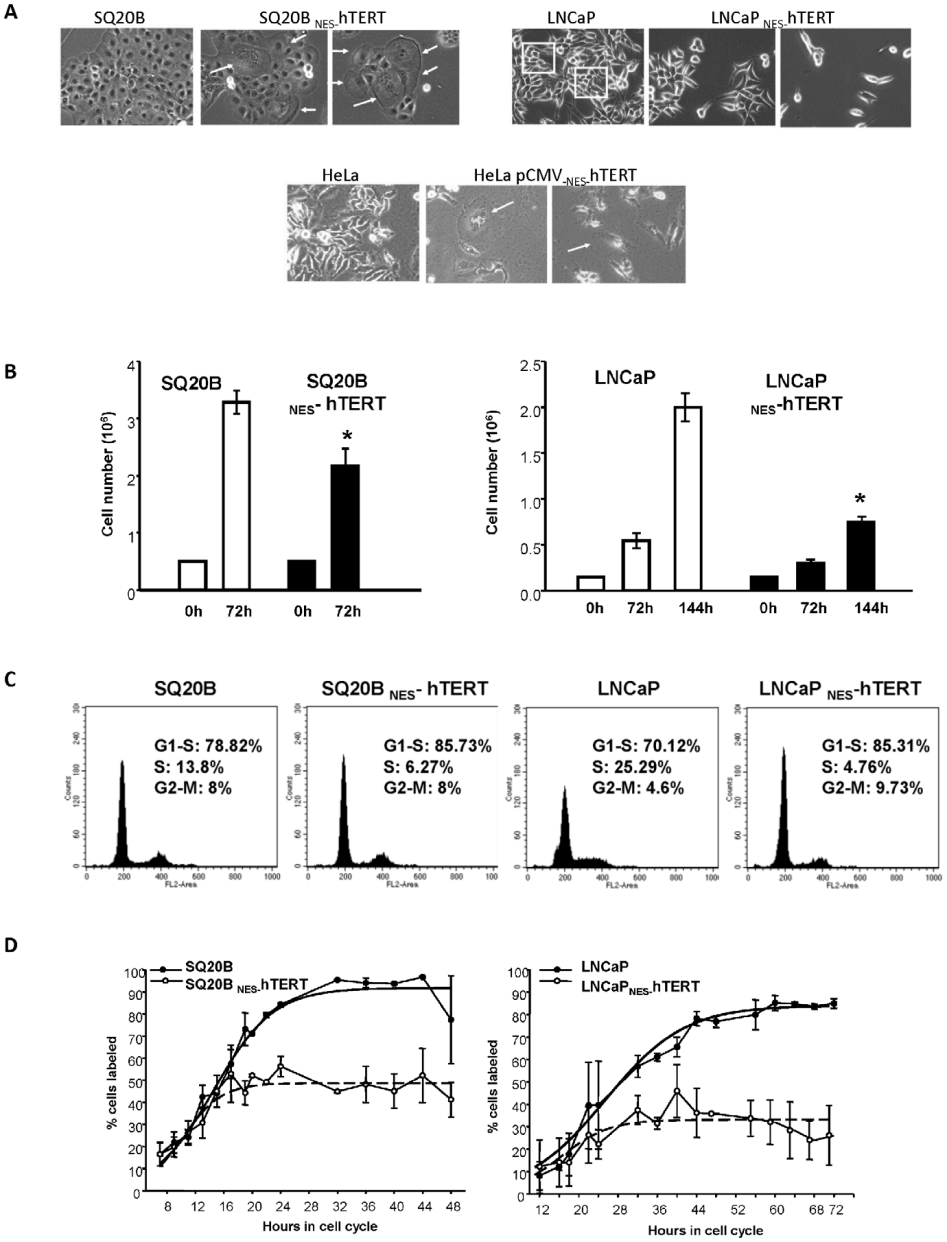
Cancer cells expressing _NES_-hTERT show changes in morphology and delays in cell cycle progression. (A) SQ20B (upper panels) LNCaP (middle panels) and their derivatives stably expressing _NES_-hTERT were plated on dishes in equal numbers and analyzed 72 hours later. Phase contrast images were taken on an Olympus IX70 microscope. Note enlarged morphology of SQ20B _NES_-hTERT and cells harboring multiple nuclei (arrows). Clustering is observed in LNCaP (see box), but not seen in LNCaP _NES_-hTERT. Bottom panels show HeLa cells that were transiently transfected with the _NES_-hTERT mutant. Images were taken 48 hours after transfections. Arrows indicate enlarged and multinucleated cells (middle) observed only upon transfection with the mutant hTERT. (B) SQ20B, LNCaP and their _NES_-hTERT derivatives were plated and allowed to grow for up to 144 hours. At various times cells were harvested and counted using a hemocytometer. In the case of LNCaP, cells were replated and counted again 72 hours later. Mean of three analyses is shown, error bars represent s.e.m. (*p≤0.05) (C) Percentage of cells in each phase of the cell cycle was calculated by flow cytometry based on PI staining. (D) Cells were serum starved overnight, then released by serum addition. Cells were labeled with [^3^H]-thymidine and analyzed at scheduled time intervals for thymidine incorporation. Mean of two independent experiments is shown, error bars are s.d.

It soon caught our attention that upon viral transduction changes in the phenotype of the cells occurred; such changes were observed while cells were still being selected. A fraction (∼30–50%) of SQ20B cells expressing _NES-_hTERT had flattened and enlarged morphology with some of these cells showing multiple nuclei (Fig. 1A, upper panels), reminiscent of the effects observed in the primary cells [19]. Unlike in the primary fibroblasts, no positive β-gal staining was observed in SQ20B cells expressing _NES-_hTERT (data not shown), suggesting that the enlarged cells were not senescent. These cells were also not apoptotic as they were neither blebbing nor detaching from the dishes (data not shown). Enlarged morphology was not observed in LNCaP cells expressing the mutant hTERT; however, while these cells tend to grow in clusters/foci irrespective of their confluence (see also ATCC), expression of _NES-_hTERT suppressed this phenotype (Fig. 1A, middle panels).

It is possible that these morphological changes simply result from the non-specific integration of the _NES-_hTERT pBabe vector. To rule out this possibility, we transiently transfected the mutant protein in HeLa cells (adenocarcinoma). HeLa cells were chosen because of the high efficiency of transient transfections (∼60%) as compared to both SQ20B and LNCaP cells (∼10–20%; data not shown) and because they too express endogenous telomerase. To assure that the cells analyzed were expressing the mutant protein, _NES-_hTERT was subcloned into the pCMV vector and either transfected alone or co-transfected with GFP; results were comparable with both approaches. Cells transfected with empty pCMV vector (+ or − GFP) were used as negative controls. As can be seen in Fig. 1A (right bottom panels), within 48 hours of expression of the mutant protein, a fraction of HeLa cells also showed enlarged and flattened morphology, which was not observed in cells transfected with the vector control (Fig. 1A, bottom left panel). These data suggest that the morphological changes observed were associated specifically with expression of _NES_-hTERT. Interestingly, no change in cell number was detected for the first 96 h following transfections with construct of the mutant protein (data not shown), while HeLa cells transduced with vector control were doubling 48 h after transfection (Fig. 1A, left bottom panel).

Next, we defined whether _NES-_hTERT altered cell cycle progression of SQ20B and LNCaP cells using three different approaches. First, we seeded similar number of cells stably expressing or not the mutant protein and followed their growth for a period of 6 days. At each time point (24, 72 and 144 hours) cells were trypsinized and counted using a hemocytometer. As shown in Fig. 1B, expression of _NES-_hTERT altered the proliferation rate of both cell types. Under these conditions, SQ20B cells underwent 1 population doubling every 28 hours while SQ20B expressing the mutant hTERT doubled every ∼36 hours. Times for doubling in the case of LNCaP cells and its _NES-_hTERT derivative were estimated at 36 and 57 hours, respectively (34 hours is the doubling time of LNCaP cells according to the vendor (ATCC)). We then monitored cell cycle progression by flow cytometry. Cells were synchronized by serum withdrawal for 16–18 hours. At 8 hours after serum addition, cells were collected and incubated with RNase A and propidium iodide (PI). The data in Fig. 1C are representative of four independent analyses; in both cell lines expression of _NES-_hTERT increased the percentage of cells in G1 while it decreased the fraction of cells in S (Fig. 1C). These data led us to quantify specifically the fraction of cells in S phase based on tritiated thymidine incorporation. Confluent cell populations were subcultured to lower density and were incubated in the presence of [^3^H]-thymidine. Movement into S-phase was monitored by autoradiography at multiple time points up to 72 hours after subculture. These experiments were reproduced two independent times and clearly showed a significant decrease in the percentage of cells in S phase upon expression of the mutant protein (Fig. 1D), consistent with the results obtained by flow cytometry. On average, by ∼20–70 hours after subculture, SQ20B and LNCaP cells expressing _NES-_hTERT had 40% and 60–80% less cells in S phase, respectively, when compared to their respective controls.

One can argue that high levels of hTERT could be driving the cell cycle effects, as previously argued [20]. However, we do not favor this hypothesis as ectopic expression of wild type (WT) or R3E/R6E hTERT, a mutant hTERT that is unable to enter mitochondria [21], had no effect on the proliferation rate of the cells (data not shown). Other groups have also ectopically expressed WT hTERT stably in various different types of cancer cells and no defects in cell cycle progression were reported [22], [23]. Taken together, the data in Fig. 1 show that expression of _NES-_hTERT efficiently and rapidly delays progression of SQ20B and LNCaP cells through the cell cycle.

### Overexpression of _NES-_hTERT in skin and prostate cancer cells decreases colony formation potential *in vitro*

**Figure 2:**
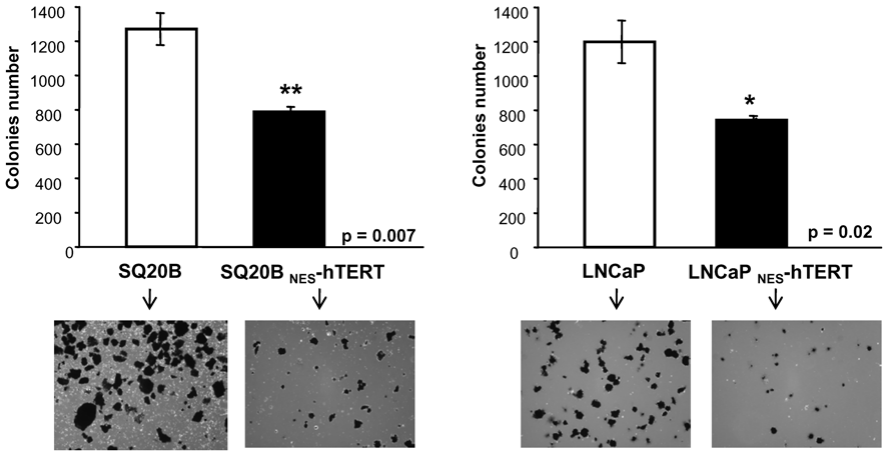
Anchorage-independent growth is diminished by expression of _NES_-hTERT. 5×10^3^ cells/well of SQ20B, LNCaP and their _NES_-hTERT derivatives were grown in soft agar for up to 3 weeks. Colony growth was evaluated every week and colonies counted based on crystal violet staining. Graphs show results from colonies counted at 1 week when individual colonies, especially in the control cells, were still easily distinguishable. Colonies were scored by two independent observers. Data shown are the average of two independent experiments done in triplicates. Bars represent mean ± sd. Representative images of the plates are shown below each graph.

A number of transformations are required for cells to become tumorigenic, including increased growth rate and ability to grow in an anchorage-independent manner [22]–[24]. The ability of transformed cells to form colonies in soft agar is a useful in vitro predictor of tumor formation in vivo [24]. We sought to investigate whether the observed effects on proliferation rate after introduction of _NES_-hTERT in skin and prostate cancer cells would impact their ability to form colonies in soft agar. To this end, we plated equal number of SQ20B, LNCaP and their respective _NES_-hTERT derivatives in triplicates in soft agar and allowed them to grow for up to 3 weeks; colonies were counted every week using crystal violet stain. Given the high amount of colonies formed in weeks 2 and 3, particularly in the controls, we scored growth after 1 week of growth in which individual colonies were still easily distinguishable. Results were reproduced two independent times. Upper panels on Fig. 2 show the number of colonies formed, lower panels show representative images of the plates. In both cancer cell types, introduction of _NES_-hTERT significantly decreased the number of colonies formed, suggesting decreased tumorigenic potential of those cells compared to the respective empty vector-expressing controls.

### Expression of _NES_-hTERT does not alter the endogenous levels of telomerase enzymatic activity but increases the levels of telomeric and extra-telomeric DNA damage

**Figure 3:**
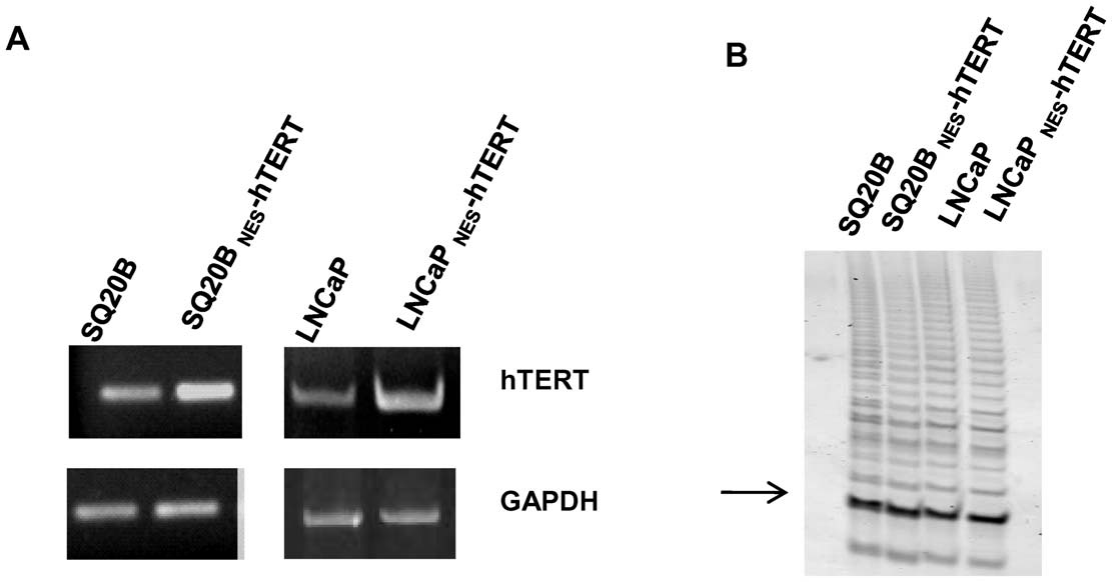
Expression of _NES-_hTERT does not alter endogenous levels of telomerase enzymatic activity. (A) Levels of hTERT RNA were gauged by RT-PCR. GAPDH was amplified and used as loading control. (B) 100 ng of total cell extracts were used to perform the TRAP. Arrow indicates internal control of the assay. Positive and negative controls are not shown.

Ectopic expression of a catalytically inactive mutant hTERT that behaved as a dominant negative was shown to efficiently inhibit telomerase enzymatic activity, leading to telomere erosion and decreased proliferation of various cancer cell types. Increased spontaneous apoptosis, decreased colony growth in soft agar and diminished tumor formation in nude mice were also observed [9], [17]. Although _NES-_hTERT is enzymatically active in vitro, it is unable to elongate telomeres in vivo [19]. Thus, it is possible that in the telomerase-positive SQ20B and LNCaP cells, _NES-_hTERT behaves in a dominant negative manner, ultimately leading to the decreased proliferation rate noted above (Fig. 1). To test this possibility, we analyzed levels of hTERT mRNA and telomerase activity in whole cellular extracts of cells expressing or not _NES_-hTERT using, respectively, RT-PCR and the telomeric repeat amplification protocol (TRAP). As expected, RNA levels of hTERT were increased in the cells ectopically expressing the mutant (Fig. 3A). However, no changes in telomerase enzymatic activity were observed by expression of the mutant protein as judged by TRAP (Fig. 3B), indicating that _NES-_hTERT does not exert a dominant negative effect upon the endogenous protein at least in terms of enzymatic activity.

In telomerase negative fibroblasts, expression of _NES-_hTERT leads to telomeric and extra-telomeric DNA damage [19]. Thus, another possible explanation for the decrease in proliferation rate is that _NES-_hTERT induces DNA damage in the cancer cells, which in turn slows down their ability to progress through the cell cycle. We tested this hypothesis by evaluating the presence of the phosphorylated form of the histone H2A variant, γH2AX, and 53-binding protein 1 (53BP1). We also detected DNA damage directly at telomeres by immuno-fluorescence in situ hybridization (immuno-FISH) in single cells, mtDNA damage was assessed by gene-specific quantitative PCR (QPCR) [25]–[27].

**Figure 4:**
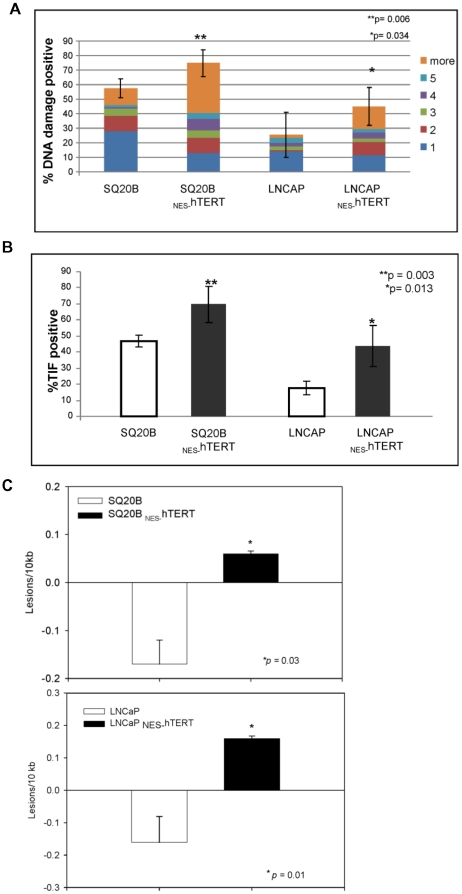
Expression of _NES_-hTERT increases nuclear and mitochondrial DNA damage in skin cancer and prostate cancer cells. (A) Cells were immunostained with antibodies against γH2AX and against 53BP1. DNA was counterstained with DAPI. Graph shows percentage of cells positive for both γH2AX and 53BP1 foci and the number and size of foci per cell (represented by the different colors according to the graph labeling). Bars are mean ± s.d. (B) ImmunoFISH staining to visualize simultaneously DNA damage foci and telomeres. DAPI was used to counterstain DNA. Graph shows percentage of DNA damage foci localized at telomeres (TIF) per single cell. (C) mtDNA integrity was analyzed by QPCR in three independent experiments. Graph show estimated lesion frequency ± s.e.m.

A form of histone H2A phosphorylated at serine 139 (S139 of γH2AX) is essential for efficient recognition of DNA double strand break (DSB) sites. Hundreds or thousands of γH2AX molecules generate nuclear foci that can be found at the site of each incipient DSB by immunostaining with antibodies to γH2AX [28]–[30]. 53BP1 is activated as part of the DNA damage response (DDR) and also form foci [28], [29], [31]. We performed single cell analysis in SQ20B, LNCaP cells and their respective _NES_-hTERT derivatives as we previously described [19], [29]. Cells were scored as having DNA damage if they were positive for both γH2AX and 53BP1 foci. Number and size of foci detected in each single cell were quantified and are represented in Fig. 4A. In both cell lines the amount of foci was significantly increased by expression of _NES-_hTERT (*p* = 0.006 for SQ20B and 0.034 for LNCaP cells). It is noteworthy that expression of the mutant protein led to a significant increase in cells presenting larger foci. For instance, the number of SQ20B _NES-_hTERT cells showing 6 foci or more was 3-fold higher than in SQ20B control cells (*p* = 0.004) (Fig. 4A).

Next, we evaluated levels of telomere-induced foci (TIF), which have been described as DNA damage foci presented at telomeric sites. TIF can arise by gradual telomere erosion due to continuous cell proliferation or by stochastic telomeric DNA damage [31]–[34]. We adopted a protocol that we previously described [29], and the number of TIF positive cells was calculated based on the total number of cells analyzed. A cell was considered TIF-positive when 50% of greater of DNA damage co-localized with telomeres. As shown in Fig. 4B, the levels of TIF-positive cells were significantly increased by expression of the mutant hTERT. Indeed, TIF-positive cells increased about 2-fold in the LNCaP background while in SQ20B this enhancement was less pronounced (Fig. 4B). In SQ20B cells, the basal level of TIF was high: about 45% of the cells were TIF-positive. Such high basal level of telomere damage was unexpected since these cells express endogenous telomerase that presumably maintains their telomeres. However, introduction of _NES-_hTERT led to a further increase in TIF that was detected in about 70% of all cells analyzed (Fig. 4B, bars on the left).

Finally, we analyzed mtDNA integrity by QPCR, as we previously showed that expression of _NES-_hTERT was associated with mitochondrial dysfunction, including loss of mtDNA integrity in primary fibroblasts. Such effects were intimately linked to the detected nuclear DNA damage at telomeric and extra-telomeric sites [19].

Assuming that damage is randomly distributed, QPCR allows an overall picture of the integrity of the genome under study [25]–[27]. The assay measures integrity of DNA using long PCR targets, in this case 8.9 kb in length, which is about 2/3 of the entire mtDNA. Given identical conditions, DNA from control and treated samples amplify differently depending on the number of lesions present on the template by the time of the reaction. For example, DNA from cells exposed to UV amplifies less than DNA from the respective non-treated control [35]. The amount of damage can be expressed as number of lesions per 10 kb assuming a Poisson distribution of lesions on the template. Presence of lesions reflects that the sample of interest amplifies less than its control, while negative number of lesions can be observed when the DNA of a given sample amplifies better than its respective control. To monitor possible changes in mtDNA copy number, a short fragment of the mitochondrial genome is also amplified in order to normalize the data. Sensitivity limit of the technique is 1 lesion/10^5^ bases (for more details on the assay, see references [25]–[27] and [35].

The data in Fig. 4C reflect the average ± s.e.m. of 3 independent analyses. Basal levels of lesions in the controls were calculated based on the average amplification of the control samples of each cell line, which was then used as a reference to compare each individual control (for more details see the Methods section). As expected, in both cellular backgrounds expression of _NES-_hTERT significantly increased basal levels of mtDNA damage (Fig. 4C), suggesting that mitochondrial dysfunction is also amplified in the cancer cells by expression of the mutant protein.

Taken together, the data presented in Fig. 4 show that expression of _NES-_hTERT in the telomerase-positive SQ20B and LNCaP cells leads to DNA damage at telomeric and extra-telomeric sites, which are not caused by a decrease in the levels of active enzyme in the nucleus but may be associated with dysfunctional mitochondria.

### _NES-_hTERT increases sensitivity to genotoxic stress in skin cancer cells

Expression of telomerase has been associated with modulation of cell death induced by genotoxic agents. Sensitization, promotion and no effects on apoptosis and/or necrosis have been reported, which seem to rely on the type of cells under study, the genotoxic agent used and, particularly, on the length of the telomeres [17], [36]–[45]. Given the significant increase in basal levels of nuclear and mtDNA damage observed upon expression of the mutant hTERT, we investigated whether the cells would be further sensitized to genotoxic stress. For these experiments, we selected SQ20B cells, which are known to be highly radioresistant both in terms of DNA damage and loss of proliferative capacity [46], [47].

**Figure 5:**
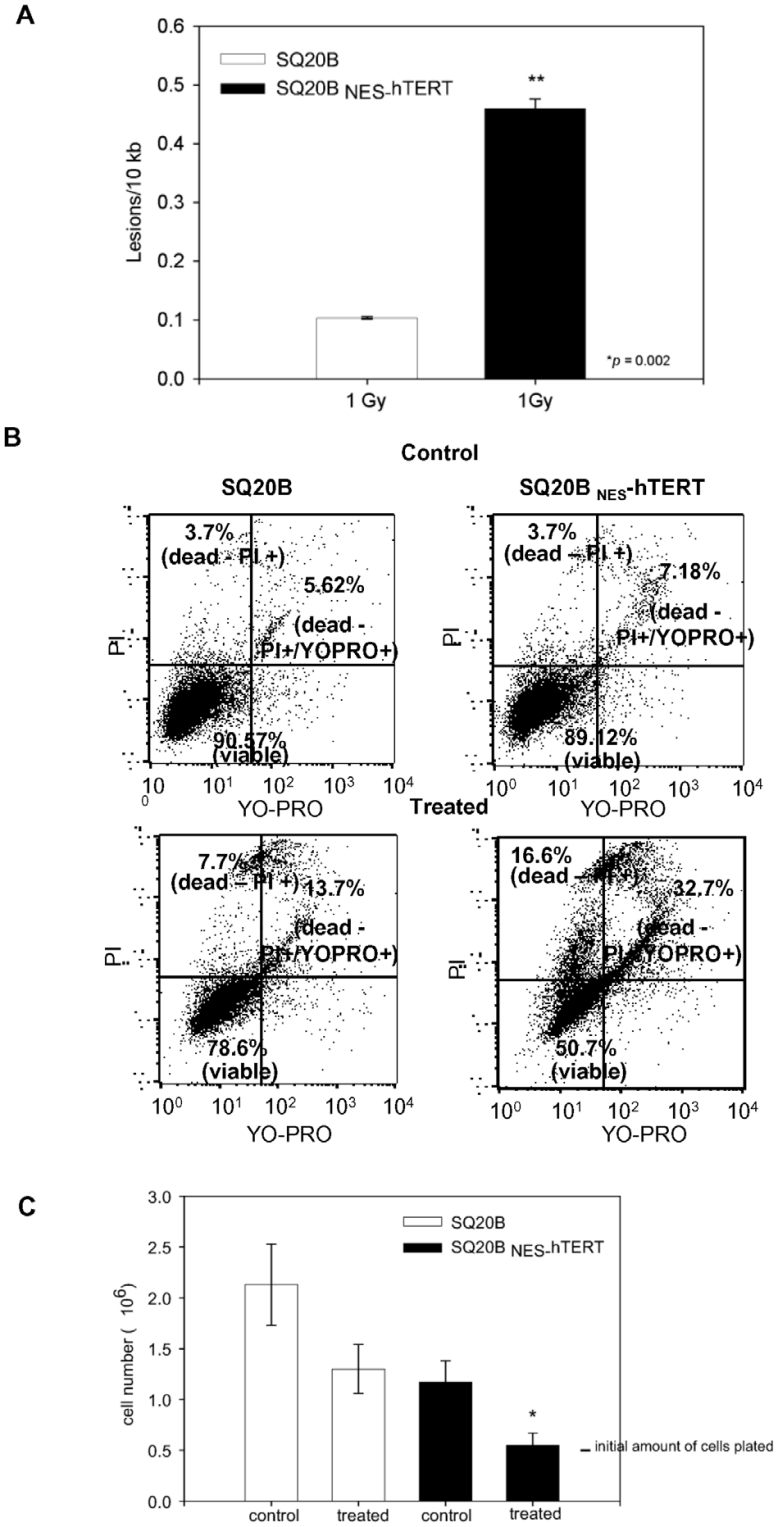
_NES_-hTERT sensitizes skin cancer cells to genotoxic stress. (A) Nuclear DNA damage was estimated in SQ20B and its _NES-_hTERT derivative immediately after exposure to 1 Gy of gamma radiation using QPCR. Results represent the average of three independent experiments ± s.e.m. (B) Cells were treated with 200 µM of H_2_O_2_ for 60 minutes and allowed to recover for 24 hours in conditioned medium. At this point, cells were harvested and the number of apoptotic, dead and viable cells was evaluated by flow cytometry using PI and YOPRO-1. Results are representative of three independent experiments. (C) The same amount of viable cells (500,000) were replated after the H_2_O_2_ exposures and their growth rate was followed for 2 weeks. The number of cells was counted using a hemocytometer at 24 hours and every time cells became confluent thereafter. As the number of treated SQ20B _NES_-hTERT did not change in the following 2 weeks, only data for 24 hours post-treatment are shown. Results are mean of three independent experiments ± s.e.m. (* p≤0.05).

In a first set of experiments, we exposed the cells to ^137^Cs γ-rays. SQ20B cells and its _NES-_hTERT derivative were plated at equal numbers and were enriched in G1 for 48 hours prior to irradiation by maintenance in the confluent state. This is important because cells in different phases of the cell cycle differ in their radiation sensitivity [48]. Cells were exposed to 1 Gray (Gy) of γ- radiation (0.65 Gy/min), and we analyzed nuclear DNA (nDNA) damage by QPCR immediately after the exposure by monitoring integrity of a 13.5 kb fragment of the β-globin gene [25]–[27]. Results presented in Fig. 5A clearly demonstrate a significant increase in the amount of γ-ray-induced DNA damage in SQ20B _NES-_hTERT cells. Whereas in control SQ20B cells, 1 lesion is observed in every 50 kb of the genome, the level of damage detected in SQ20B _NES_-hTERT cells is 5-fold greater, translating to 1 lesion every 10 kb of double stranded DNA (Fig. 5A).

Next we determined whether the cells would be sensitized to other types of stresses. To this end, we exposed them to hydrogen peroxide (H_2_O_2_) and analyzed cell death by flow cytometry. We chose H_2_O_2_ because of our experience with this oxidative stressor; experiments were performed as described by us previously [21], [49], [50]. Briefly, equal number of SQ20B cells was seeded 16 hours prior to H_2_O_2_ exposures. Cells were treated with 200 µM H_2_O_2_ for 60 minutes in basal medium in the absence of FBS and were harvested either immediately following exposure to H_2_O_2_ or allowed to recover for 24 hours in conditioned growth medium. At both points, the amount of dead and apoptotic cells was scored based on propidium iodide uptake (PI) and YOPRO-1 staining. YOPRO-1 is a green-fluorescent dye that detects specifically apoptotic cells [51]–[55]. Cells were analyzed by flow cytometry to quantify with greater confidence the percentage of viable, dead and apoptotic cells. As no significant changes in these parameters were observed immediately after the H_2_O_2_ treatment, the data presented below relate to the 24 hours recovery point.

Figure 5B illustrates experiments that are representative of 3 independent analyses. A large increase in the amount of YOPRO-1 and PI-positive cells was observed in the treated SQ20B background expressing the mutant hTERT (Fig. 5B). Quantification of the number of viable, dead and apoptotic cells revealed that while a 2-fold increase in the number of dead cells (either PI-positive only or PI and YOPRO positive) was observed in SQ20B 24 hours after the treatments, this increase was about 5-fold in SQ20B expressing _NES-_hTERT (Fig. 5B). No significant differences in the basal rate of dead/apoptotic cells were detected when comparing non-treated SQ20B with the mutant-expressing derivative (Fig. 5B, upper panels).

To look for long-term effects of the treatments, we then followed the proliferation rates of the control and treated SQ20B and SQ20B _NES_-hTERT for 2 weeks after the H_2_O_2_ exposure. Equal numbers of viable control and treated cells (0.5×10^6^) was plated and their number counted using a hemocytometer in the first 24 hours and every time cells reached 100% confluence thereafter. While controls and treated SQ20B doubled in number at least once in the first 24 hours, no change in cell number was observed in treated SQ20B _NES-_hTERT (Fig. 5C). Remarkably, these cells remained quiescent for 2 additional weeks when they finally started doubling (data not shown). These results are particularly intriguing considering that SQ20B harbor a mutated p53 that is unable to induce the G1-S checkpoint upon DNA damage [46], [47].

Taken together, the data shown in Fig. 5 demonstrate that expression of _NES-_hTERT is able to sensitize SQ20B to γ-radiation and to oxidative stress caused by H_2_O_2_.

## Discussion

In the present study we showed that introduction of _NES-_hTERT, a mutant that is defective in nuclear-cytoplasmic shuttling, into squamous carcinoma (SQ20B) and prostate cancer (LNCaP) cells results in significant delays in cell cycle progression, decreased proliferation rate and anchorage-independent growth (Figs 1, 2). These effects were not associated with decreased endogenous telomerase enzymatic activity since expression of the mutant hTERT did not alter TRAP activity (Fig. 3). We also observed increased DNA damage in telomeric and extra-telomeric sites, and higher number of mtDNA lesions under normal conditions upon expression of _NES-_hTERT (Fig. 4). Remarkably, the hTERT mutant sensitized SQ20B cells that are otherwise highly resistant to ionizing radiation-induced DNA damage and to cell death induced by H_2_O_2_ (Fig. 5). Taken together, our data suggest manipulating the NES of hTERT or telomerase's subcellular shuttling as novel and efficiently means to counteract tumor cell growth.

### _NES-_hTERT affects cell cycle and tumorigenicity of cancer cells *in vitro* without behaving as a dominant negative mutant

We have recently shown that expression of _NES-_hTERT in primary cells leads to premature growth arrest with accompanying morphological and genetic changes involved in cellular senescence [19]. In the present study, we found similar changes in SQ20B and LNCaP cells after expression of the mutant wherein a significant decrease in the rates of cell cycle progression and proliferation were observed, which was accompanied by alterations in cell morphology (Fig. 1). However, no markers of senescence were evident in the cells (data not shown). Further, significant decrease in colony formation in soft agar was observed after introduction of the mutant (Fig. 2), which likely resulted from decreased proliferation rate and increased doubling time.

The lack of a complete growth arrest and the absence of senescence markers in the cancer cell lines upon expression of _NES-_hTERT were not surprising because activation and maintenance of cellular senescence rely on the function of the tumor suppressor p53, its downstream effector p21^waf1^ and in the activation of p16/pRb [56]. These signaling pathways are defective, respectively, in SQ20B and LNCaP. While SQ20B harbors a mutated p53 unable to transactivate p21^waf1^, the p16 gene in LNCaP is subject to aberrant methylation, leading to transcriptional inactivation and functional loss [47], [57]. Given these observations, it is tempting to speculate that activation of both pathways is required for a complete growth arrest provoked by expression of _NES-_hTERT. This hypothesis is supported by our previous observations that expression of _NES-_hTERT in a SV40-transformed cell line in which both p53 and p16 are disrupted had no effects on cell cycle regulation [19].

It is yet unclear how _NES-_hTERT could impair the cell cycle of SQ20B and LNCaP cells. One obvious possibility is that the mutant competes with the endogenous protein, ultimately leading to decreases in telomerase enzymatic activity that affects telomere maintenance. This concept of dominant negative effect regarding hTERT mutants is not new and has been shown to effectively halt proliferation rate, as well as both in vitro and in vivo tumorigenicity of various cancer cell types [9], [17]. However, these observations do not explain our data, since expression of _NES-_hTERT did not alter the total levels of endogenous telomerase activity as judged by the TRAP (Fig. 3). In addition, the effects of the dominant-negative mutants previously reported were linked to telomere shortening (due to the lack of telomere elongation) and increases in basal apoptotic rate. Not surprisingly, the shorter the telomeres were prior to expression of the dominant-negative mutants the faster growth defects and cell death appeared [9], [17]. We have not measured specifically telomeric length in the cells, but results with immuno-FISH suggest that on average telomeres of LNCaP cells were longer than in SQ20B cells, which had fairly short telomeres prior to expression of the mutant. Even with initial differences in their telomeric lengths, the proliferation defects observed upon introduction of _NES-_hTERT were detected in the same time frame (that is under the selection process) making it unlikely that they relied on telomere shortening. In addition, no increases in basal cell death rates were observed (Fig. 5 and data not shown).

It is possible that _NES-_hTERT behaved as a dominant negative in terms of subcellular shuttling, impeding the nuclear export of the endogenous protein and ultimately leading to the same effects as expression of _NES-_hTERT in telomerase-negative cells [19]. This possibility may also explain why cells expressing the mutant hTERT have decreased mtDNA integrity (Fig. 4). We previously found that _NES-_hTERT is not present in mitochondria, which was associated with a high degree of mitochondrial dysfunction [19]. Our unpublished results show that a fraction of endogenous hTERT is mitochondrial in both SQ20B and LNCaP cells (Gordon and Santos, in preparation). Complete lack of mitochondrial hTERT in SQ20B and LNCaP cells could potentially drive the degree of mitochondrial impairment, which is already noteworthy in cancer cells [58], to a limit that impacts cell cycle regulation either through increased reactive oxygen species (ROS) and DNA damage, changes in oxygen utilization and/or energy production. We did not monitor markers of mitochondrial function *per se* but since the integrity of the mtDNA is intimately associated with proper mitochondrial function [45], [59] it is likely that mitochondria are further impaired in the cancer cells expressing _NES-_hTERT. More studies are required to better understand this issue.

The high levels of DNA damage present in the cells upon expression of the mutant protein (Fig. 4) may also play a role for the cell cycle delays observed. One can envision that in the presence of such high degree of damaged DNA, the cells need to slow down in their progression through the cell cycle in order to repair the damage [33]. Although the total levels of DNA foci were already high in control cells, expression of the mutant increased the level to a degree that was likely above the threshold that the cells could ‘efficiently’ tolerate. In this regard, it is worth noting the significant increase in the number of larger foci/cell after expression of _NES-_hTERT (Fig. 4).

The flow cytometry analyses using PI revealed that the cells were accumulating in the G1-S transition (Fig. 1). p53 is a master regulator of DNA damage signaling involved particularly in the G1-S checkpoint [60]. While the involvement of p53 could explain the results in LNCaP cells, it does not apply for SQ20B cells that harbor a defective version of the protein. It is likely that the latter (or both cell lines) activate a yet different set of genes to trigger the G1-S delay. One likely candidate is p38 MAPK, which can contribute to the G1-S checkpoint in response to diverse stimuli in a p53-independent manner. Interestingly, the contribution of p38 MAPK to the G1-S transition is particularly evident upon damage by ROS and telomere-related senescence [61]. Further studies are required to define which signaling pathway(s) involved in cell cycle regulation is modulated in cancer cells by expression of _NES-_hTERT.

The number of TIF positive cells significantly increased upon expression of _NES-_hTERT. Although the number of TIF doubled in LNCaP _NES-_hTERT compared to its control, a more modest increase was observed for SQ20B cells. The latter may reflect the fact that SQ20B _NES-_hTERT cells had many very short telomeres that did not hybridize well (or at all) with the telomeric probe used for the assay (data not shown). Irrespective, one intriguing observation from this study was the high basal degree of TIF in SQ20B cells even prior to expression of the mutant protein (Fig. 4B). This is unexpected given these cells express endogenous telomerase that is presumably functional at telomeres and thus competent to sustain their replicative potential. These data may indicate that the levels of telomerase that allow cell proliferation, at least in this cancer cell line, are not the same required for maintenance of a ‘functional’ telomeric structure. In accordance with this assumption, Cesare and co-workers [62] recently reported that immortalized human cell lines lacking wild-type p53 spontaneously show many telomeres with a DNA damage response (DDR). In telomerase-positive cells, DDR was associated with low telomerase activity and short telomeres that were proposed to represent an intermediate configuration between the fully capped and uncapped (fusogenic) states [62].

### Manipulation of hTERT subcellular localization may provide a new therapeutic approach in cancer treatment

We show here that overexpression of _NES-_hTERT renders SQ20B cells more sensitive to DNA damage caused by ionizing radiation and to cell death-mediated by oxidative stress. More unexpected, strong but transient growth arrest (for about 2 weeks) was observed in the viable cells that were re-cultured after H_2_O_2_ exposures (Fig. 5). These results were very surprising owing to the well-established radioresistance of SQ20B cells [47]. However, they may reflect that only a small fraction of the mutant-expressing cells were in S-phase, which is associated with increased resistance to ionizing radiation given the conformation of the DNA [63]. Alternatively, it has been shown that GSH levels are lower in G1 and higher in S [64]–[66], which could explain the resistance of SQ20B to cell death mediated by H_2_O_2_ while increasing sensitivity of the mutant expressing cells. Finally, the additional stress provoked by the exogenous damaging agents upon already heavily damaged DNA may have pushed the cells towards death. Associated with this is the presence of very short telomeres in the mutant-expressing cells, which are known to be associated with genomic instability [43]. More work is certainly required to understand exactly how the _NES-_hTERT mutant can sensitize cells to genotoxic damage and which kind of cell-tissue type would positively respond to such intervention.

## Materials and Methods

### Cell Culture

SQ20B cells were grown in Eagle's minimal essential medium (Cellgro) supplemented with 10% fetal bovine serum (Gibco/Invitrogen), 1% penicillin and streptomycin (Invitrogen) as previously described [50]. LNCaP and HeLa cells were obtained from ATCC. Cells were grown in Dulbecco's modified Eagle high glucose medium (Gibco/Invitrogen) supplemented with 10% fetal bovine serum and 1% penicillin and streptomycin.

### Plasmids and viral infections

Retroviral pBabe vector empty or carrying wild type or hTERT mutants, and the pCMV vectors used were described earlier [19], [21], [50]. Transient and stable transfections were performed as described previously [21], [50]. Images shown in Figure 1 were acquired with an inverted Olympus IX70 microscope (80X) with MicroFire digital camera.

### Cell growth

Equal number of SQ20B and LNCaP cells and their respective _NES_-hTERT derivatives were plated in 75 cm^2^ flasks and followed for up to 144 hours. At 24, 72 and 144 hours cells were harvested by trypsinization and total number of attached cells was counted with a hematocytometer.

### Cell cycle analysis

*Flow cytometry:* SQ20B, LNCaP and their _NES_-hTERT mutant cells were serum starved overnight (16–18 hours), then released from serum starvation for 8 hours by addition of 10% FBS into the medium. Cell cycle analysis by flow cytometry with propidium iodide (PI, Molecular Probes) was performed as described earlier [19] using a BD Biosciences FACSCalibur flow cytometer. DNA content analysis was performed by Modi Fit *LT* (Verity Software House).

*[^3^H]thymidine labeling*. SQ20B, LNCaP and their _NES_-hTERT derivatives cells were serum starved for 48 hours, then trypsinized and plated on 35 mm dishes (3×10^4^ cells per dish) in 2 ml of the medium containing 2 µCi/mL of [^3^H]-thymidine (specific activity 20 Ci/mmol) (PerkinElmer LAS, Inc), and 10% FBS. At regular intervals, duplicate dishes were rinsed with PBS, fixed with ethanol, and subjected to autoradiography. To determine labeling indices, a minimum of 1000 cells/dish were scored. The use of this continuous labeling technique allows precise determination of G_1_ delays [67]. The percentage of cells in S-phase was determined as described [68].

### Anchorage-independent growth in soft agar

SQ20B, LNCaP and their respective _NES_-hTERT derivative cells were seeded on six-well plates at a density of 5×10^3^ cells in 2 ml of 0.3% agar layered onto 0.6% agar. Cells were grown for up to 3 weeks, at 37°C in a humidified 5%CO2/95% air chamber, and colonies were stained with crystal violet and counted every week. Medium was replaced every 4–5 days or as needed. Colonies were scored in a blinded fashion by two independent observers.

### DNA integrity by gene-specific quantitative PCR

QPCR was followed as described previously [25]–[27]. The analyzed cells were derived in two independent viral infections. To define the basal level of damage in the control cells, the relative amplification of all control samples was averaged and used as a reference to compare each individual control. Damage on the mutant-expressing counterpart was estimated relative to the non-_NES-_hTERT control. For more details on the assay see references [25]–[27].

### DNA damage foci

Cells were grown on coverslips for at least 48 hours prior to immunostaining; they were then processed and stained with anti-γH2AX and 53BP1 antibodies as described previously [19]. Images were acquired using a Zeiss Axiovert 200 fluorescence microscope equipped with ApoTome.

### TIF analysis by ImmunoFISH

Cells were processed and stained with anti-γH2AX and anti-53BP1 antibodies as described above and protocol for TIF followed as described [19], [29]. Cells were mounted as described above and analyzed by UV microscopy using a Zeiss Axiovert 200 fluorescence microscope equipped with ApoTome. Images were acquired as z-stacks spaced 0.4 µm apart using a 100X lens with 1.4 optical aperture.

### Telomeric Repeat Amplification Protocol (TRAP)

Total protein extracts (100 ng per sample) were assayed for TRAP using TRAPeze kit (Chemicon) according to manufacturer's instructions and with some modifications [50].

### H_2_O_2_ treatment, cell viability and apoptosis

Cells were plated in 60-mm dishes (0.5×10^6^ cells per dish) 16 hours prior to the experiment. H_2_O_2_ experiments were performed as described earlier [21], [50]. Cells were either collected immediately after the treatment or allowed to recover for 24 hours in conditioned medium. Cell viability and apoptosis were analyzed respectively with PI and YO-PRO-1 (Invitrogen) by flow cytometry. Cells were treated for 1 h with 200 µM H_2_O_2_ and were allowed to recover for 24 hours when both control and treated cells were harvested. Cells were washed twice with 1 ml of PBS, and then stained with a final concentration of 2.5 µM of YO-PRO-1 and 1 µg of PI for 20 minutes on ice. After this period, cells were analyzed using a BD Biosciences FACSCalibur flow cytometer. Percentage of apoptotic, dead and living cells were scored using the Cellquest pro (BD Biosciences) software. Results represent mean of at least three independent experiments.

### Irradiation

Cells were enriched in G_1_ for 48 hours prior to irradiation by maintenance in the confluent in serum-free medium for 48 hours. The cells were irradiated with 1 Gy of γ-rays (0.65 Gy/min) from a ^137^Cs source in a ventilated irradiator (J.L. Shepherd, Mark I, San Fernando, CA). Immediately prior to irradiation, the flasks with the cells were placed on a rotating platform to ensure uniform exposure dose per dish. After irradiation cells were collected for DNA integrity analysis by QPCR. Results represent mean of three independent experiments.

### Statistical analysis

Unpaired Student *t*-test was performed to calculate statistical significance (P≤0.05).
